# Actin-Tethered Junctional Complexes in Angiogenesis and Lymphangiogenesis in Association with Vascular Endothelial Growth Factor

**DOI:** 10.1155/2015/314178

**Published:** 2015-03-25

**Authors:** Dimitar P. Zankov, Hisakazu Ogita

**Affiliations:** Division of Molecular Medical Biochemistry, Department of Biochemistry and Molecular Biology, Shiga University of Medical Science, Seta Tsukinowa-cho, Shiga, Otsu 520-2192, Japan

## Abstract

Vasculature is present in all tissues and therefore is indispensable for development, biology, and pathology of multicellular organisms. Endothelial cells guarantee proper function of the vessels and are the original component in angiogenesis. Morphogenesis of the vascular system utilizes processes like cell adhesion, motility, proliferation, and survival that are closely related to the dynamics of actin filaments and actin-tethered adhesion complexes. Here we review involvement of actin cytoskeleton-associated junctional molecules of endothelial cells in angiogenesis and lymphangiogenesis. Particularly, we focus on F-actin binding protein afadin, an adaptor protein involved in broad range of signaling mechanisms. Afadin mediates the pathways of vascular endothelial growth factor- (VEGF-) and sphingosine 1-phosphate-triggered angiogenesis and is essential for embryonic development of lymph vessels in mice. We propose that targeting actin-tethered junctional molecules, including afadin, may present a new approach to angiogenic therapy that in combination with today used medications like VEGF inhibitors will benefit against development of pathological angiogenesis.

## 1. Introduction

Endothelial cells (ECs) in mature vascular system are quiescent, nonproliferating (with some exceptions, e.g., uterus) heterogenic population. The endothelium generated by a single layer of ECs separates the blood and lymph from other components of the vessel wall and serves wide variety of functions, specific not only for the vascular bed but also for the tissue they populate [1, 2]. ECs are the first component of blood vasculature that is formed in the embryo by differentiation of mesodermal precursor cells angioblasts (process defined as vasculogenesis, VG), thus creating the primary capillary plexus [3]. Subsequently, the embryonic vasculature evolves from the existing vessels by remodeling (termed as angiogenesis, AG) [4]. In contrast, lymphangiogenesis (LAG) starts with migration, proliferation, and differentiation of ECs pool residing in cardinal vein [5, 6].

Intercellular junctions between the adjacent ECs and between ECs and surrounding non-EC wall components (e.g., pericytes) maintain the organization of EC layer and vessel integrity. Their function is beyond just mechanical support involving at least inhibition of ECs proliferation and neovasculogenesis in mature vessels as well as regulation of ECs gene expression and survival [7]. Adhesive machinery of ECs includes adherens and tight junctions and focal adhesions [8], all associated with intracellular F-actin network. Morphogenesis of vasculature relies on processes like cell adhesion, motility, and proliferation that inevitably include the actin cytoskeleton and associated junctional molecules, making the majority of these complexes a requisite of VG, AG, and LAG [9–14].

In this review we focus on the involvement of actin-associated molecules at the junctional apparatus in AG and LAG and, in particular, afadin, an adaptor protein with multiple roles in cellular physiology [15]. Small GTP-binding proteins (GTPases) Rap1 and RhoA are discussed in the context of afadin signaling. The role of GTPases related to actin cytoskeleton organization and AG is beyond the scope of this paper. The interested readers may refer to a number of outstanding publications [16–18]. We have selected this particular view on vascular development, because those adherent complexes are deeply interwoven with the signaling of the “prime switches” of AG: vascular endothelial growth factors (VEGFs) and their receptor-tyrosine kinase VEGF receptors [19], which makes them appealing target for pro/antiangiogenic therapy.

## 2. Afadin in the Pathways Controlling AG and LAG

**Figure 1:**
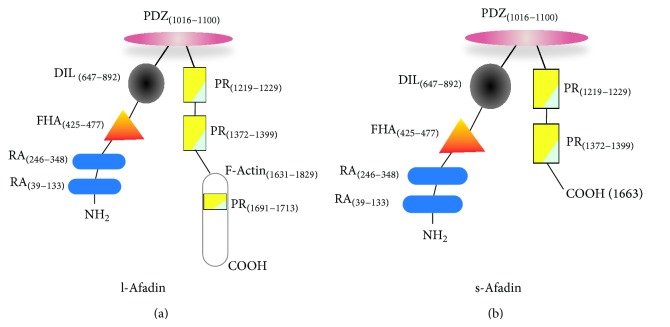
Anatomy of afadin molecule. The modular structure of l-afadin (a) and s-afadin (b) is schematically shown. Numbers in parentheses indicate the first and last amino acid of the structural domains. RA: Ras associated domain; FHA: forkhead associated domain; DIL: dilute domain; PDZ: postsynaptic density, Drosophila disk large tumor suppressor, zonula occludens-1 domain; PR: proline rich domain; F-actin: F-actin binding domain. PDZ domain interacts with nectin molecules.

Afadin is an adaptor protein discovered in 1997 by Mandai et al. and holds two RA (Ras association), a FHA (forkhead-associated), a DIL (dilute), a PDZ (postsynaptic density, Drosophila disk large tumor suppressor, zonula occludens-1), three PR (proline-rich), and F-actin structural domains (Figure 1) [20]. Two isoforms are described at present: l-afadin and s-afadin. s-Afadin truncates the C-terminal F-actin and the third PR domains. l-Afadin is expressed ubiquitously, whereas s-afadin is expressed mainly in the nerve tissue [21]. F-actin and PDZ domains link actin filaments and Ig-like transmembrane junctional proteins nectins, respectively. Resulting cell-cell adhesion assembly is crucial for establishment and part of adherens and tight junctions in epithelia, fibroblasts, and ECs [15, 22]. In addition, afadin functions independently of nectins to promote cell movement and neuronal physiology [23–26]. Due to the multitude of interacting domains and fundamental role of cell-cell junctions for tissue organization [27], afadin is involved in various biological phenomena ranging from embryonic development to cancer progression. Complexity of those processes creates a broad field of constantly increasing information of afadin roles [28–32].

Physiological AG, the formation of blood vessels from existing ones, occurs not only in the embryo but also in postnatal life (e.g., in uterus, during wound healing). Pathological AG accompanies some chronic inflammatory diseases (e.g., rheumatoid arthritis), cancer, and atherosclerosis [4, 33, 34]. During physiological AG, there is fine-tuned balance between stimulating and suppressing factors in order to maintain vascular and tissue integrity and assure effective vessel formation [35]. Pathological AG results in disorganized, abnormal vasculature with disturbed regulation [4].

Undeniably, the prime molecular machinery that stimulates VG and sprouting AG is comprised of VEGF and VEGF receptor in ECs [4, 33, 34, 36]. VEGF receptor interacts with key ECs adhesion molecules (e.g., VE-cadherin, neuropilin, and integrins), guides tip ECs to VEGF signal, and activates a myriad of intracellular signaling during all phases of AG. Genetic inhibition of VEGF or VEGF receptor in mice prevents successful vessel formation and cause embryonic death [37–39].

**Figure 2:**
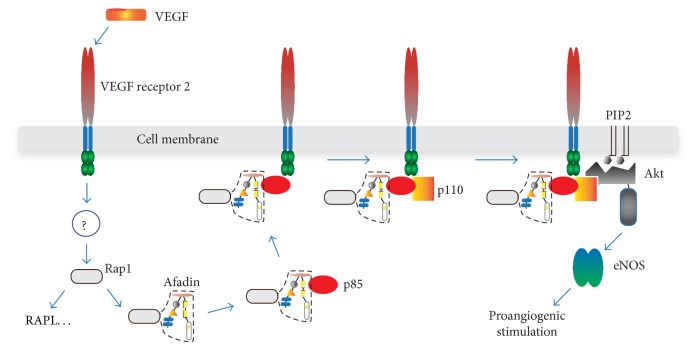
Proposed function of afadin in the VEGF or S1P receptor signaling during AG. In HUVECs, activated Rap1 (by still unknown mechanism) binds and recruits afadin to cell membrane where the complex between VEGF receptor, afadin, Rap1, and sequentially p85 and p110 subunits of PI3K assembles. Activated PI3K phosphorylates Akt and downstream signaling follows. Similar events occur after S1P receptor activation (not drawn). In addition, Rap1 stimulates different proteins (e.g., RAPL) that may contribute to proangiogenic signal.

VEGF signaling is also critical for tumor AG. At present the most extensively applied medication in human cancer treatment is VEGF inhibitors [40]. One of the downstream targets of activated VEGF receptor is Rap1 GTPase that is also indispensable for the vessel formation [41]. In epithelial cells Rap1 associates with afadin and recruits epithelial (E)-cadherin to adherens junctions [42]. Understanding of the partners of activated Rap1 in ECs had not been extensive when we investigated Rap1-driven mechanisms in VEGF and sphingosine 1-phosphate- (S1P-) induced AG [43]. By studying VEGF- or S1P-stimulated human umbilical vein ECs (HUVECs) and conditional knockout (cKO) mice with endothelial-specific afadin gene disruption, we found that (i) in HUVECs, intracellular localization of afadin was Rap1-dependent and colocalization of activated (GTP-containing) Rap1 and afadin was observed in the cell-cell contacts and the leading edge of polarized moving cells; (ii) afadin or Rap1 knockdown in HUVECs reduced VEGF- or S1P-stimulated capillary-like network formation in Matrigel and 3D gels, suppressed migration and proliferation of HUVECs, and increased the number of apoptotic cells; (iii) equivalent to the epithelial cells, Rap1 and afadin played key roles in accumulation of adherens and tight junction proteins since absence of afadin or Rap1 in HUVECs removed the fluorescent signal in the cell membrane for nectin-2, VE-cadherin, claudin-5, and junctional adhesion molecule A; (iv) in VEGF- or S1P-stimulated HUVECs, afadin and Rap1 controlled specifically phosphorylation of Akt and endothelial nitric oxide synthase (eNOS) but not extracellular signal-regulated kinase or p38. The subsequent experiments revealed that afadin is essential for the interaction between phosphoinositide 3-kinase (PI3K) regulatory subunit p85 and VEGF or S1P receptors. That interaction recruits catalytic subunit p110 of PI3K and Akt/eNOS phosphorylation follows (schematically drawn in Figure 2). Akt/eNOS signaling is proangiogenic and downstream of VEGF and S1P in ECs [44, 45].

We have also found that, in endothelial afadin cKO mice, postnatal development of retinal vessels is initially delayed, that small vessels network decreased, and that VE-cadherin staining in ECs became discontinuous. Even in heterozygous KO mice, recovery of blood flow and neoformation of capillary networks after hind limb ischemia were diminished compared to control mice. Those* in vivo* data validated the vital role of afadin in postnatal AG. Moreover, afadin is involved in maintaining epithelial and endothelial barrier function [46–48], migration of cancer cells [29, 32], elongation and lumen formation of the developing nephron [49], and modulation of integrin levels in epithelial cells [50]. All those findings provide evidence that afadin is one of the main participants in the mechanisms of cellular adhesion, motility, and proliferation, processes implicated in AG but also in a broad variety of other physiological and pathological events.

VEGF receptor-Rap1 activation signal in ECs is transmitted not only to afadin/Akt but also to p42/44 ERK1/2 and p38 MAPK [51]. Those tyrosine kinases are important for ECs proliferation and actin cytoskeleton remodeling and are absolutely required for placental AG [52, 53]. In Rap1b-deficient mice AG is disturbed resulting in embryonic and perinatal mortality [54]. The mechanisms for the defective AG comprise ECs dysfunction (decreased activation, migration, and proliferation) that coincides with lower levels of p42/44 ERK1/2 and p38 MAPK in ECs. Another downstream target of Rap1 in ECs is RAPL, protein that associates with GTP-bound Rap1 to activate integrins [55]. This pathway has proved essential in angiogenic sprouting, ECs migration (HUVECs), adhesion, and* in vivo* neovascularization (hind limb ischemia model in mice) [56]. The role of RAPL in AG is attributed to inside-out integrin signaling: RAPL promotes *β*1 integrin affinity, thereby stimulating ECs adhesion and migration (for the role of integrins in AG and LAG, see the next section of this review).

**Figure 3:**
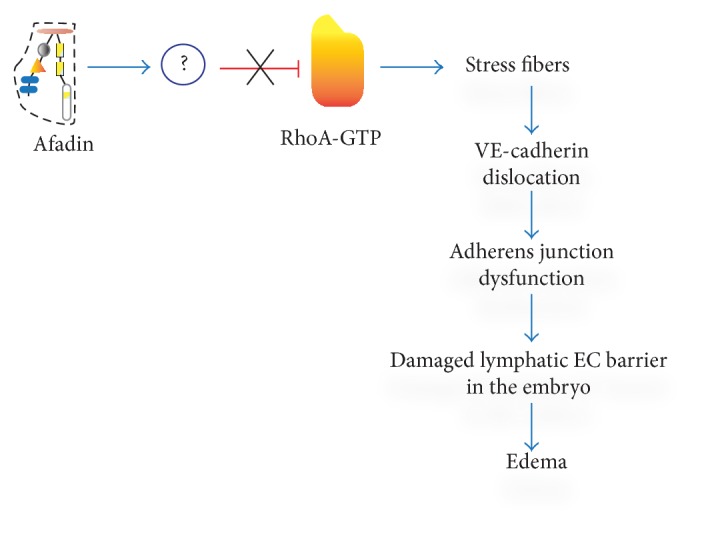
Sequence of regulatory steps leading to damage of lymph endothelial barrier in afadin cKO mouse embryos. In the absence of afadin-mediated inhibition of RhoA activity, actin stress fibers are formed. Thick actin filaments alter the cell shape, dislocate VE-cadherin from cell membrane, compromise adherent junctions, and damage lymph EC barrier. This results in generalized edema and embryonic death.

The mice with EC-targeted deletion of afadin [43] were born in a significantly reduced ratio (3.6% versus 25% expected), showing that afadin is also important for embryonic AG. That observation became the topic of our investigation [57], in which we found that most of the endothelial afadin cKO mouse embryos died at embryonic day (E) 16.5. Until E13.5, no detectable dissimilarities between cKO and control embryos could be observed, but at E14.5–E16.5, cKO mice developed diffuse subcutaneous edema and dot-like skin hemorrhages. Series of immunofluorescence experiments demonstrated that lymphatic vessels in the skin of afadin cKO embryos were largely dilated and that lymphatic endothelium exhibited defect in VE-cadherin staining but preserved ability to differentiate and proliferate. To investigate afadin-induced dysfunction of lymphatic endothelium, human dermal blood and lymphatic microvascular ECs (BMVECs and LMVECs, resp.) were used as a model. Knockdown of afadin in LMVECs triggered cell-shape alteration, disorganization of cell-cell contacts, reduction of VE-cadherin staining, and formation of thick F-actin fibers at the cells periphery. All of these effects were not produced in BMVECs. Afadin-associated F-actin/VE-cadherin rearrangements in LMVECs depended on RhoA activity: GTP-bound (active) RhoA was increased in afadin-knockdown LMVECs and dominant negative RhoA mutant rescued the phenotype in LVMECs. Thus, afadin stabilizes adherens junctions/VE-cadherin by suppressing RhoA activity and stress fibers formation to maintain EC barrier in embryonic lymphatic vessels (Figure 3). Actin fibers organization is reported as condition that defines the localization of VE-cadherin in cell junctions. Actin depolymerization or hyperpolymerization has been shown to decrease VE-cadherin in ECs and compromise endothelial barrier [58]. Contraction of cortical actin-myosin cytoskeleton in ECs stimulated by Rho GTPase has similar effect [59]. Actin bundles in ECs formed after cAMP/Epac/Rap1 stimulation anchor VE-cadherin and strengthen cell-cell adhesions, the effect that results in reduction of endothelial layer permeability [60, 61].

Our reports described above complement the knowledge about signaling in embryonic LAG and postnatal AG. Lack of afadin in lymphatic ECs of the mouse embryo does not impede vessel morphogenesis but compromises intercellular junctions of ECs. This effect is secondary to F-actin stress fiber synthesis and VE-cadherin dislocation. Contrary to adhesion structures in blood ECs, lymphatic ECs have “button-like” intercellular contacts that facilitate the transport of intercellular fluids [62]. This functional specialization may also include unique RhoA regulation by afadin. In addition, RhoA-dependent actin rearrangement and disruption of cell-cell adherens junction resemble the mechanism exploited by thrombin to damage barrier function in lung ECs [47]. Afadin/Rap1 complex was essential for recovery of cell-cell contacts and actin network in lung ECs.

Participation of afadin in the signaling downstream of VEGF and S1P receptors as well as its involvement in tubulogenesis and apoptosis might focus attention on this molecule in the context of tumor AG and cancer therapy. Afadin is also important antiapoptotic guard in embryogenesis, and platelet-derived growth factor (PDGF) receptor binds PI3K regulatory subunit p85/afadin in a similar way as shown for VEGF and S1P receptors to activate Akt [63].

## 3. Other Actin-Associated Adhesion Complexes in relation to AG and LAG

Many transmembrane adhesion molecules in ECs and their intracellular partners that link the molecular assembly to actin filaments contribute to the complex sequence of events in the process of VEGF-induced AG. Studies with cKO mice that eliminate the function of selected molecule in ECs demonstrated the individual role of excluded junctional component in AG or LAG and revealed its unique phenotype.

### 3.1. VE-Cadherin

VE-cadherin communicates through its cytoplasmic segment with *β*-catenin, plakoglobin, and p120-catenin. *β*-Catenin and plakoglobin interact with *α*-catenin that anchors (although recently questioned [64]) VE-cadherin to actin cytoskeleton [65, 66]. p120-Catenin stabilizes VE-cadherin by preventing its endocytosis [67]. VE-cadherin deficiency in ECs of mice created by cKO technique has caused embryonic lethality at E9.5 because of increased EC apoptosis and defective AG [68]. VG was not disturbed, but lack of VE-cadherin stopped the remodeling of primary capillary plexus. Some vessels of the embryos had narrow or no lumen, whereas others were dilated. Throughout the vasculature, ECs were detached from the basement membranes, disconnected each other, and could be seen in the lumen of the vessels. Those observations resulted from a failure to form the EC survival complex amid VEGF, VE-cadherin, *β*-catenin, and PI3K, missing Akt phosphorylation and thus inactive antiapoptotic machinery [69].

VE-cadherin is crucial for the functional integrity of endothelial layer [70] and contributes to all phases of AG. Upon initiation of VEGF-mediated AG and activation of VEGF receptor, VE-cadherin is target for Src phosphorylation, a modulation that leads to adherens junction disassembly and increased permeability of ECs. Simultaneously, molecular complex consisting of integrin *α*v*β*3, syndecan-1, and insulin-like growth factor-1 receptor is activated. These molecular events facilitate early adhesion and migration of ECs in VEGF-induced AG [71]. During sprouting neovascularization, the tip and stalk ECs are recognized in the developing branches [72]. It has been found that tip and stalk ECs are not static but they exchange their positions and phenotypes in the process of elongation of the vessel [73]. VE-cadherin dynamics under contrasting control of VEGF and Notch signaling drives this ECs behavior and is necessary for the coordination of physiological AG. VE-cadherin mobility is lost in certain pathology, for example, cancer [74]. VE-cadherin is important for establishing the functional vessels by inhibiting VEGF-signaling (p44/p42 MAPK) and ECs proliferation after ECs contact and adherens junction formation [75].

### 3.2. *β*-Catenin

Mice embryos with inactivated *β*-catenin in ECs die at E11.5–E13.5 [10]. VG and early AG developed, but after E9.5 vessels showed lumen irregularities and hemorrhage. Vitelline vessels had smaller diameter. The pattern of the vasculature of primitive neural plexus was defective, and the placenta was less vascularized compared to control mice. ECs had morphological changes, impaired junctions, and abundant fenestrations. In cultured ECs of *β*-catenin cKO mice, immunofluorescence of plakoglobin was increased, *α*-catenin decreased, and desmoplakin was found at EC contacts but not at those of control mice. The shift of molecular composition of junctional proteins in the *β*-catenin cKO mice has led to the authors' interesting hypothesis: lack of *β*-catenin forces creation of extra VE-cadherin/plakoglobin complexes and plakoglobin/desmoplakin/vimentin interaction analogous to that in* complexus adhaerentes* in lymphatic vessels [76]. In addition, proliferation of cultured ECs stimulated by VEGF receptor was not inhibited by confluency in the absence of *β*-catenin [75]. ECs express an array of Frizzled/Lrp (low-density lipoprotein receptor-related protein) receptor complexes, a target of Wnt (Wingless and Int-1) ligand [77]. Canonical Wnt signaling includes *β*-catenin transcriptional activity through interaction with T-cell factor (Tcf)/Lef transcription factors [78]. Wnt canonical system is involved in embryonic AG [79] and the vascular phenotype of *β*-catenin cKO mice may partially be a result of Wnt signal failure. Interestingly, the constitutively active *β*-catenin mutant in ECs also impairs embryonic AG because of overactivity of Notch-related pathways [80].

### 3.3. p120-Catenin

p120-Catenin stabilizes membrane localization of VE-cadherin by preventing endocytosis [81]. Deletion of p120-catenin in ECs of mice is also embryonically lethal starting at E12.5 (40% of mutated embryos) [11]; however, some of them survive without obvious abnormalities. p120-Catenin deletion causes defects in microvasculature after E9.5; reduced microvascular density, disorganized vascular networks with impaired branching and blind-ending vessels, and hemorrhages were found in the brain and other organs. Pericytes recruitment, VE-cadherin, and N-cadherin expression were decreased. Cultured ECs from p120-catenin cKO mice exhibited proliferation deficiency.

### 3.4. Integrins

Integrins are large family of adhesive proteins that interact with extracellular matrix components and some adhesion molecules in the cell membranes and are critical for cell motility and survival [82]. They are heterodimers of *α* and *β* subunits that upon activation cluster to form focal adhesions and assemble intracellular signaling molecules that are linked to actin cytoskeleton and specific cellular functions. In addition, integrins regulate VE-cadherin/catenin complexes during cell movement [83]. A vast number of reports show the importance of particular integrins in AG [8, 84, 85]. Integrin *α*v*β*3 is coreceptor of VEGF receptor in AG [86] and null or cKO mice (ECs) for several integrin subunits like *α*v, *α*3, *α*5, *β*1, and *β*3 demonstrate their obligatory role in AG [87–89]. Deficiency of those subunits causes severe defects in vascular development: disturbed vessel organization, defective tubulogenesis, hemorrhage, vessel wall rupture, and embryonic death. Intracellular adaptor molecules linked to integrin dimers also control AG. Deletion of talin-1, which directly associates with *β* subunit of integrins and mediates coupling to actin, in ECs of mice produced severe defect in AG with diffuse hemorrhages and disrupted vascular trees, leading to embryonic death [90]. Kindlin-2, the newly discovered *β* subunit partner important for integrin activation [91], is also critical for AG. In mice, heterozygous deletion of kindlin-2 resulted in formation of immature vessels in implanted tumors. ECs from those mice showed reduction of integrin *β*3-dependent adhesion, migration, and tube formation. Another key component of integrin activation, focal adhesion kinase (FAK), induced tumor AG [91].

Integrin *α*9*β*1 appears to have specific role in LAG. Integrin *α*9-null mice suffered insufficiency of the valves in collecting lymph vessels, developed chylothorax, and inexorably died by postnatal day 12 because of respiratory failure [92]. The valves had malformed leaflets due to missing integrin *α*9*β*1/fibronectin interaction that is necessary for the leaflets matrix organization [93]. Integrin *α*9*β*1 has unique property of binding directly VEGF to promote ECs adhesion and migration [94].

ECs in lymphatic microvasculature have specialized junctions that apparently reflect specific functionality of lymph capillaries [62]. Those “button-like” structures form discontinuous line of adhesions that allow easy flow of interstitial constituents into the lumen. During embryonic development ECs in lymphatic capillaries have continuous intercellular junctions that evolve to the “buttons” soon after birth [95]. The molecular composition of the junctions in lymphatic ECs is mixture of adherens and tight junctional proteins (VE-cadherin, occluding, claudin, afadin, etc.). Contrary to the role of those proteins in AG, importance of actin-associated junctional molecules in LAG (except partially for afadin) remains to be elucidated.

## 4. Necessity for Clarifying the Role of Similar Molecules in Developmental and Pathological AG and LAG

Pathological AG and LAG are considered dysregulated processes utilizing similar molecular repertoire as AG and LAG during embryogenesis [4]. However, there is still little knowledge about the specificity of signaling mechanisms in pathological AG and LAG. Available reports have demonstrated altered functionality of certain angiogenic molecules like placental growth factor (PIGF), VEGF receptor-1, and VEGF-B in the context of diseased state [96–98]. Those pathology-associated investigations of the key angiogenic molecules such as VEGF ligands and receptors are the actual basis for understanding and eventually discovering the targets for antiangiogenic therapy. Actin-related junctional molecules are probably not exception and they may have additional/changed functionality in diseased conditions: for example, VE-cadherin in neoplastic vascular ECs expresses epitope that normally is obscured and this molecular segment allows specific targeting of tumor ECs [99]. Promising objective for antiangiogenic therapy especially in cancer is integrin *α*v*β*3, essential molecule for tumor AG. At present, several groups of pharmacological inhibitors of integrin *α*v*β*3 are in the different stages of testing and the results show favorable effects in preclinical and clinical settings [100].

## 5. Conclusion

AG and LAG are attractive targets for influencing pathological conditions that present with excessive/abnormal vascular proliferation (chronic inflammation, cancer) or ischemia (atherosclerosis). The growth of vessels in AG demands fundamental processes of cell biology and morphogenesis: cellular adhesion, migration, proliferation, and survival. All of these functions utilize as essential effector actin filaments and associated junctional molecules that assure intercellular or cell-matrix communication. As a part of the same machinery, actin-tethered molecular complexes appear to be highly integrated, and they cooperate to regulate organization or disorganization of particular adhesion structure. Furthermore, the main switch of AG, VEGF signaling, is intimately dependent on these molecular complexes. Because of these properties, molecular assemblies including VE-cadherin, integrin, and nectin as well as actin may be considered as (co)targets of VEGF-related pro- or antiangiogenic therapy.

Afadin functions downstream of angiogenic signals translated by VEGF and S1P in ECs and also may represent target for modulating AG. Afadin has multiple roles in cellular processes, which may broaden the effects of its inhibition. Not only physiological AG but also pathological AG (e.g., neovascularization in tumors) might be affected by modulating the afadin-related signals. However, there are many unanswered questions at present to justify this strategy: compromising intercellular adhesions may promote metastasis in cancer, whereas dysfunctional actin-related machinery might have opposite effects by preventing cell adhesion and promoting apoptosis; fundamental roles of cell junctions most probably will require targeted approach, delivery of active substances only to the diseased location; mechanisms involving afadin in pathological AG could have altered effects. Nevertheless, the proposed hypotheses might be worth testing in translational studies with hope that it will help in reducing the resistance to VEGF receptor inhibitors in VEGF-induced (or other) pathological AG.
